# An evaluation of a student-led career profiling project to support the exploration of a career in general practice and other specialties

**DOI:** 10.3399/BJGPO.2022.0002

**Published:** 2022-08-10

**Authors:** Hannah Gyekye-Mensah, Arabella Watkins, Joseph Wenden, Imogen Horn, Jemimah Beardwood, Melvyn Jones, Emma Metters

**Affiliations:** 1 St George’s University of London, London, UK; 2 Musgrove Park Hospital, Taunton, UK; 3 Croydon University Hospital, London, UK; 4 St George’s University of London, Institute of Medical & Biomedical Education, London, UK; 5 Department of Primary Care and Population Health, UCL, London, UK

**Keywords:** career choice, career guidance, general practice, medical students

## Abstract

**Background:**

Choosing medical careers is complex but the undergraduate period is formative. St George’s University of London (SGUL) students called for greater careers information.

**Aim:**

To develop and evaluate students’ careers resources.

**Design & setting:**

A quality improvement student–staff project at SGUL, UK.

**Method:**

A ‘Plan-Do-Study-Act’ (PDSA) cycle was completed. For the ‘Plan’ element, students’ career intentions and information preferences were surveyed. For the ‘Do’ element, video interviews with clinicians and infographic posters were produced and published on SGUL’s virtual learning environment. For the ‘Study’ element, feedback questionnaires were thematically analysed using Kirkpatrick’s framework. For the ‘Act’ element, the model was rolled out across SGUL programmes.

**Results:**

In the ‘Plan’ stage, 79 students ranked interest in specialties, with general practice being the second most popular. Students were unconfident in how to pursue careers and wanted more information. For the ‘Do’ element, 13 careers videos and infographics were created for 10 specialties. The ‘Study’ questionnaire showed changes across three of the four levels in Kirkpatrick’s model of evaluation of training. Level 1 (Response): students found resources helpful and accessible. Level 2 (Learning): students reported increased understanding of careers. Level 3 (Transfer): students planned using checklists and made career comparisons by specialty. Level 4 (Results): students’ career choices were not demonstrated, but there were tentative proxy measures such as copying and modelling career routes and choices. ‘Act’ involved rolling out and regularly updating resources.

**Conclusion:**

This PDSA model enabled development of resources by students mapped to students’ needs. Changes were demonstrated in relation to students’ response, learning, and transfer, with tentative suggestions of impact on career choice.

## How this fits in

Advice on medical careers at the undergraduate level is key. There has been little published on effective ways of delivering and evaluating the impact of careers resources for medical students. Careers talks can be usefully supplemented with structured video interviews and infographics. There appear to be useful opportunities to shape students’ interest in general practice careers at the undergraduate level.

## Introduction

Choosing a medical career is complex and the undergraduate period is a formative one, in which potential choices may be reinforced or discarded.^1^ The process of selecting a career often begins before medical school^2^ and continues beyond graduation. Junior doctors apply for specialty training during the foundation programme,^3^ meaning time to explore specialties beyond medical school is limited,^4^ with a scarcity of information available in the foundation years.^5,6^

Various reviews identify student demographics or medical school factors that influence career choices.^4^ Decisions are known to be partially moulded by students’ undergraduate experiences of a given discipline.^1^ The culture of the medical school is also an important influence on students’ choices.^7,8^ There is, however, uncertainty about which factors are important, and to what extent undergraduate factors can influence longer-term decisions about careers; for example, in primary care.^9^

The General Medical Council (GMC) has specified that medical schools should provide career advice,^10^ but this advice is no longer mandated in the current GMC ‘Outcomes for graduates’.^11^ The 2008 Tooke report identified careers guidance as vital to the NHS’s recruitment strategy for junior doctors.^12^

Specifically, NHS recruitment for general practice is a major issue.^13^ In 2016, Health Education England (HEE) and Medical Schools Council (MSC) produced the Wass report, outlining how medical schools could improve GP recruitment.^13^ Suggested interventions included helping students to make informed career decisions (recommendation 15 in the Wass report),^13^ and this careers resource is now available online from HEE.^14^

Relevant, informative, and accessible careers advice at medical school is key,^15,16^ but how best to effectively deliver this to students is not known. This study found limited published evidence for the role of active careers interventions at medical school and how effective these might be.^1^ ^1,4,17–22^

This article aimed to describe how SGUL’s careers advice provision was updated and evaluated.

## Method

SGUL is a UK medical school with a 5-year undergraduate course and a 4-year graduate entry programme.^23^ SGUL’s National Student Survey highlighted the need for increased careers information.^24,25^ In response to this, a Plan-Do-Study-Act (PDSA) intervention cycle was undertaken. The PDSA framework is widely used in health care, and its very pragmatic approach allows testing of small-scale interventions and rapid assessment of their impact.^26^ This approach was chosen as there were no specific resources available beyond student time. For the ‘Plan’ element, students were surveyed to identify what careers resources they wanted and to inform the planning and design of new novel online careers resources. For the ‘Do’ element, video interviews with consultants, GPs, and trainees, backed by infographic posters which included specialty training information, were produced and published on SGUL’s virtual learning environment.

For the ‘Study’ element, this careers intervention was evaluated using Kirkpatrick’s 4-level model of evaluation of training.^27^ The ‘Act’ element was determined by the response to these initiatives, which included institutional presentations. Kirkpatrick’s model is widely used as an evaluative framework in health care and education. Level 1 is the reaction level: how participants react to the training. Level 2 looks at learning: what participants have learnt as a result of the training. Level 3 identifies behaviours or activity that have changed as a result of the activity. Level 4 explores if there are results of the activity.

The careers resources were evaluated via an online feedback questionnaire which was sent to SGUL medical students. This survey was circulated to all SGUL students 3 months after the resources were launched. Quantitative questionnaire data were analysed using simple descriptive statistics, and there was a thematic analysis of qualitative data.^28^ The anonymised usage of the online video and infographic resources was captured.

The data from the follow-up survey were used to establish if there were changes at Kirkpatrick Levels 1–3. It would not be expected to see Level 4 ‘Results’ changes within the study period, as these would take several years to become apparent; the research team did, however, explore whether any change in careers intention could be observed as a proxy for Level 4 results.

The project was conceptualised as a quality improvement project, so ethics approval for the evaluation was not required. Participation in the survey was optional and anonymised.

## Results

### Plan

As part of the ‘Plan’ element, a staff–student partnership group (SSPG) was formed, consisting of six medical students, the MBBS careers tutor (a GP), and an SGUL careers consultant. Table 1 shows a reflexive statement of authors’ career stage and interests.

**Table 1.** Reflexive statement of authors’ interests

Author	Career stage	Career interests	Study role
Hannah Gyekye-Mensah^a^	HG-M is a final year medical student	HG-M is interested in general practice, medical education, psychiatry, and obstetrics and gynaecology	Involved in SSPG, developing, piloting of resources, and data collection. Involved in analysis and write-up. HG-M was lead author.
Dr Arabella Watkins^a^	AW is in F2 training in South West England	AW is interested in psychiatry and medical education	
Dr Joseph Wenden^a^	JW is in F2 training in South West England	JW is interested in trauma and orthopaedic surgery	
Dr Imogen Horn^a^	IH is an F2 doctor at Croydon University Hospital	IH is interested in sexual health and global health	
Dr Jemimah Beardwood^a^	JB is in F2 training in South West England	JB is interested in global health, medical education, and paediatrics	
Dr Melvyn Jones	MJ is a reader in general practice and portfolio GP	MJ is a practising GP and works in medical education	Involved in data analysis and write-up.
Dr Emma Metters	EM is a senior lecturer in general practice and a practising GP	EM is a practising GP and works in medical education, and is the SGUL careers lead	Principal investigator and project lead. Involved in analysis and write-up.

^a^Clinical medical students at the time of the project. F2 = foundation year 2. SSPG = staff–student partnership group.

An initial survey of medical students in November 2018 established existing knowledge of speciality training pathways, which specialities students most wanted additional information about, and the preferred format for such information. Seventy-nine responses were received, of which 65 were from clinical students. Most students declined to provide further demographic information, but of those who did (*n* = 19), most were female (*n* = 18) and on the 5-year course (*n* = 19). Overall response rates were low, but highest among clinical students (up to 9.6%).

**Figure 1.**
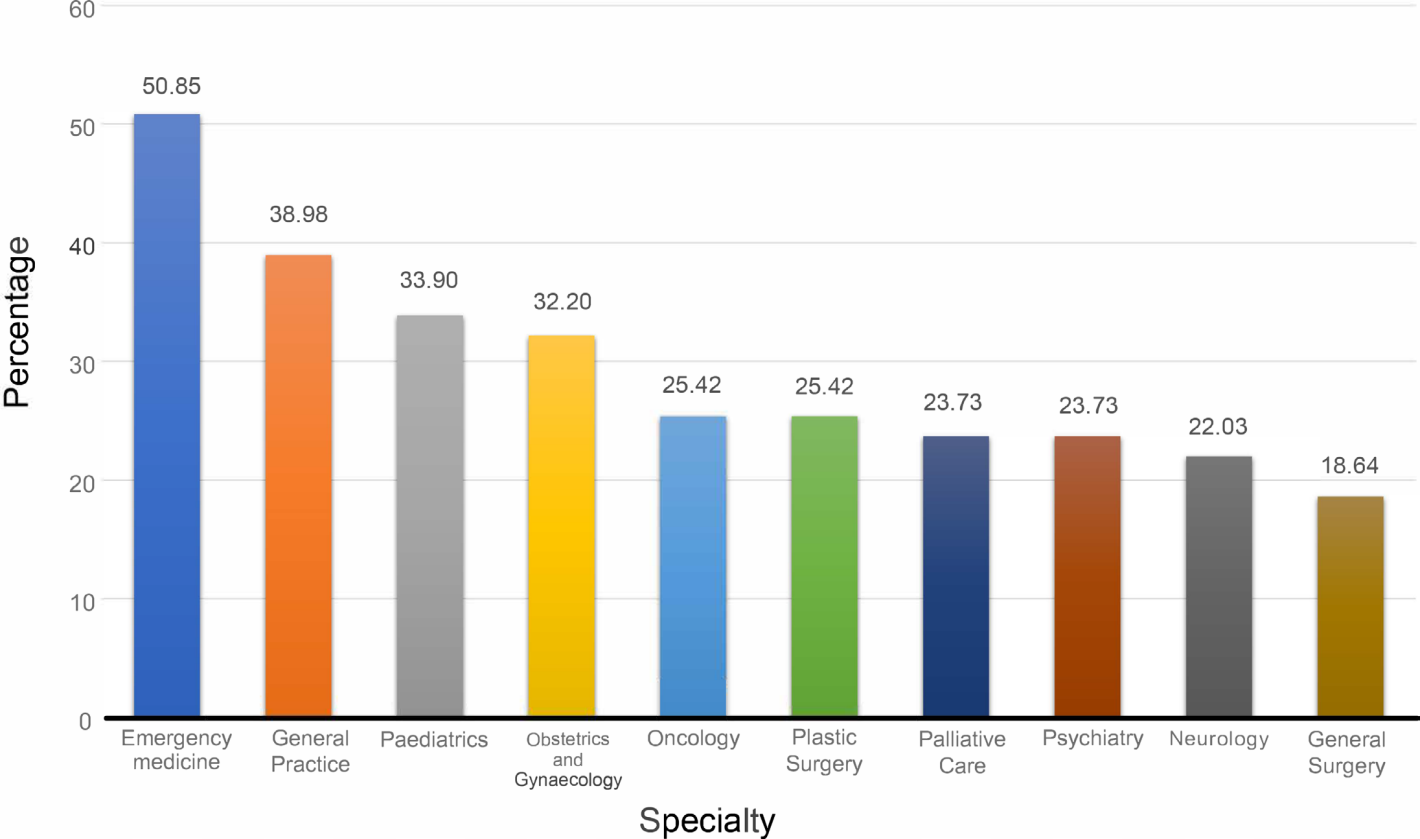
Percentage of students interested in more information, by specialty (*n* = 79).

General practice was among top scoring specialties, with 39% of all the students who responded selected general practice as a specility they were interested in as shown in Figure 1. Only 35% of the 79 students surveyed felt confident in how to pursue a career in their chosen specialty; 94% wanted more careers information. Events with speakers and interview style videos were cited as preferred formats for information delivery, followed by written information. Careers events with speakers were already delivered through the SGUL MBBS programme. In response to the survey results, the focus was put on designing and developing new careers videos and infographics in specialties prioritised by the student survey (Figure 1).

### Do: Development of the training intervention

The students from the SSPG created 13 career videos covering the 10 highest ranked specialties, with supporting infographics (see Figures 1 2) to supplement the existing careers talks.

**Figure 2.**
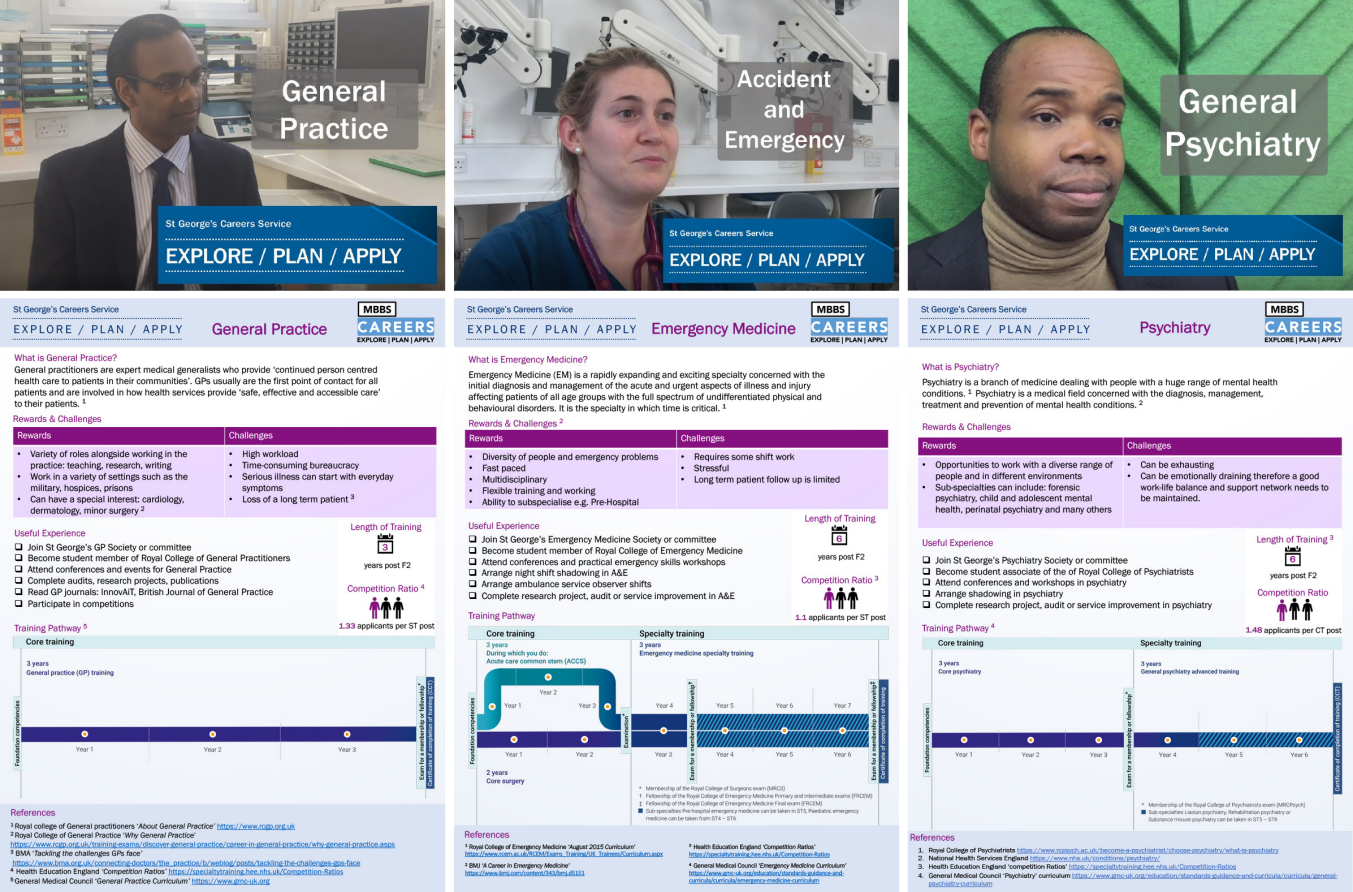
Example videos and infographics.

The project group developed an interview framework including standardised questions across specialties (Appendix 1), based on resources previously developed for the Academic Foundation Programme. Inspiring role models were identified through SGUL alumni and SSPG students’ placement experiences. Once the SSPG students had video production training, video interviews were conducted with GPs, consultants, and trainees without any piloting. Videos were edited for brevity, but interviewees could not review or change the content. The supporting infographic posters provided summary information covering training pathways, competition ratios, and further careers resources (Figure 2).

The new resources were published on the SGUL virtual learning environment and advertised via newsletters, emails, and student placements in early 2020.

### Study

An online feedback questionnaire was undertaken from February to May 2020 (overlapping with COVID-19 lockdown measures in the UK from 23 March 2020. Of the 66 responses, 68% (*n* = 45) were studying on the 5-year programme and 59% were female (*n* = 39). Moreover, the majority (*n* = 56) were in the clinical years.

In terms of resource usage, infographic resources were used more than the videos (see Table 2). The rank orders of how often a resource was used varied between infographic and video. Interpretation was limited, as some specialities had videos from both consultants and registrars. The general trend indicated hospital surgical and medical specialties attracted most interest, followed by obstetrics and gynaecology, with general practice behind these disciplines (ranked joint fifth and joint eighth in infographic and video views, respectively). This was at odds with the higher numbers of students from the initial survey wanting more information about GP careers (see Figure 1).

**Table 2.** Video and infographic usage

Specialty	Infographic		Video	
	Total views	Usage ranking	Total views	Usage ranking
Core surgical training	57	1	21	2
Plastic surgery	53	2	14	5
Emergency medicine	51	3	17	4
Obstetrics and gynaecology	43	4	19	3
General practice	41	5 (joint)	9	8 (joint)
General surgery	41	5 (joint)	11	6 (joint)
Neurology	41	5 (joint)	9	8 (joint)
Paediatrics	39	8	12 + 12^a^	1
Psychiatry	29	9	3 + 8^a^	6 (joint)
Oncology	24	10	7	10
Palliative medicine	23	11	3	11
**Total**	**442**		**145**	

^a^Consultant videos plus registrar videos.

#### Kirkpatrick Level 1: Reaction

Sixty-six students completed the feedback questionnaire, of which 85% (*n* = 56) scored the usefulness of the resources as at least 4 out of 5 (5 = most useful, 1 = least useful).

Qualitative feedback identified the importance of career information and difficulty accessing this from other resources:

*‘The only way you can learn about a speciality is to have a conversation like that with doctors, but you have very little exposure to some of those specialities in lower years, so this* […] *is very useful.’*

Students responded positively to resources and information about specific specialities:

*‘The videos were excellent and very informative about the day-to-day life of specialties.’*

They were also positive about the accessible format of information provided:

*‘Really useful and relevant information* […] *really fantastic resource.’*

Other students responded to specific information on how to progress through certain careers:

*‘Summarises all the key information and shows the structure of the training and when you move up the ranks.’*

One important consideration was the timing of the resources’ availability in relation to where students were in their careers:

*‘I wish this was shown in 1st/2nd year as it is a very useful resource.’*

Earlier provision could allow students to tailor their activities and have enough time to develop their CVs for specific careers:

*‘… focus on specific ways on how to boost your portfolio in medical school and foundation years.’*

#### Level 2: Learning

Among responders, 94% (*n* = 62) reported an increased understanding of the specialties covered by the resources. Students valued hearing clinicians’ perspectives, which they described as ‘*honest*’ and ‘*insightful*’. They appreciated understanding more about daily life and training pathways, which they did not get to explore on placements:

*‘Good to have advice for students and the drawbacks of specialties.’*

Understandably, there was a lot of uncertainty about possible careers:

*‘…* [students] *are still unsure of what they want to do in the future.’*

However, students were often focused on what it would be like to follow a specialty during the early part, rather than the whole arc of a career:

*‘Interesting to see the viewpoints of doctors in training as well as consultants.’*

*‘Trainees are more relatable.’*

#### Level 3: Transfer

There was evidence of Kirkpatrick’s knowledge ‘transfer’, as students planned using the resources as checklists and were able to directly compare specialties:

*‘Infographics are super helpful and can actually be used as a checklist.’*

*‘Really liked the videos all asking the same questions so you could compare specialities.’*

An example of transfer involved students preparing and gaining extra skills:

*‘Advice on how to prepare at medical school for certain specialties.’*

Students highlighted the difficulty of gaining these extra skills:

*‘It’s easy to say things like ‘‘get involved in research and audits’’ as advice, but this is practically impossible if you don't have any connections to clinicians.’*

and how this might be resolved:

*‘Helpful, especially the ‘‘useful experience’’ bit, may include links to other ways we can get involved?’*

Students showed an awareness of the competitive nature of entry to many specialties as hinted at in the the following quote:

*‘*[useful to have] *The competition ratios and pros and cons of each specialty.’*

They also wanted to know how they could gain an advantage over other applicants:

*‘… more ‘‘off-piste’’ opportunities which set candidates apart.’*

#### Level 4: Results

It was not possible to demonstrate Level 4 changes in terms of career choice within the time scale of this project. However, there were tentative suggestions of proxy measures of choice, such as copying career routes:

*‘Learning about different peoples’ careers and how I might be able to copy them on my own.’*

and modelling career choice:

*‘The descriptions of day-to-day life in that specialty, the drawbacks,’*

and its associated lifestyle:

*‘The daily life of a surgeon. As worklife balance is something very important for me.’*

There was realisation among students that many clinicians’ careers were unplanned:

*‘… we get the impression* […] *that every consultant had decided on their future speciality before they took even their GCSEs, but in reality, it’s much less organised than that.’*

### GP versus hospital careers

General practice appears reasonably popular in comparison with other specialties, as do other frontline specialties like emergency medicine and psychiatry (Figure 1). However, the qualitative data largely focused on careers in secondary care:

*‘It is nice to have consultants doing the video, as they have a wealth of knowledge regarding their speciality’*

Very few sought further information on primary care. However, some students are thinking about general practice, public health, or some other community-focused speciality:

*‘Knowing about what you can do with you career outside of the hospital is nice, as obviously we would struggle to know that until we are in that position.’*

The diverse student population at SGUL, in terms of graduate entry and students with caring responsibilities, is picked up in quotes suggesting priorities such as flexibility within specialties when considering careers:

*‘… students are from alternative pathways; for example, mature students, student parents. We need more insight to the realities of the different pathways and the difficulties to be faced when entering practice later on in life.’*

### Act

The project and evaluation were presented and positively received at SGUL’s education and careers forums. Plans are in place to produce resources for a wider range of specialties and distribute the model and resources across other SGUL programmes. The timing of release of resources — and to which students they are released — need consideration, to allow students opportunities to tailor their CVs by joining societies or undertaking projects, research, or audits. This rollout is the ‘Act’ element of the PDSA cycle.

## Discussion

### Summary

This SSPG at SGUL produced a bank of accessible new careers resources, which were mapped to students’ needs. General practice and other frontline specialties do seem to be of interest to students based on the initial survey, but this is at odds with national trends.^29^ The remote delivery of these resources was important during the COVID-19 pandemic, when students had reduced contact with clinicians and limited opportunities for informal career conversations.^15^

### Strengths and limitations

The positive feedback demonstrates the value of having relevant and useful resources developed by and for students. Evaluation of careers interventions is rare, and the use of quality improvement evaluation frameworks is therefore relatively novel in this field, as are data about student engagement and preferences on the mode of delivery, which are absent from the literature. These were strengths of the study and something other medical schools may consider when developing careers advice.

There are limitations to this initiative, primarily related to it being from one institution only, and therefore of limited generalisability. It was only possible to produce a limited number of new resources, and these were prioritised by student preference, as indicated in the first survey. What students prioritised would likely be a complex mix of personal interests and gaps in available knowledge or resources, hence their idiosyncratic selections omitting some common general medical specialties like cardiology (perhaps addressed by curriculum exposure) and ranking niche specialties like plastic surgery (not covered by SGUL's curriculum). Challenges in the analysis were presented by the limited response rate and demographic data on students’ sex,^30^ graduate status, or age,^31^ which are known predictors of career choice. Establishing students’ prior career preferences may have helped further evaluate the impact of the resources, but in the interests of survey brevity this was not done.

The impact of careers interventions can take several years to become apparent. What’s more, there will be multiple factors involved in influencing career choice which are beyond the scope of this quality improvement project, so any suggestions of Level 4 changes or proxy changes in this study must be highly tentative. The aim of this project was, however, to improve students’ knowledge and understanding about careers, rather than to direct them towards a specific career.

### Comparison with existing literature

There has been little published on effective ways of delivering and evaluating the impact of careers resources for medical students. As evaluation of careers interventions is rare, this study’s findings about student engagement and preferences regarding the delivery mode are novel.

### Implications for research and practice

The project model provides a framework for institutions to produce a student-focused careers resource that appears to have a positive impact on students’ understanding and engagement with career planning, including in the speciality of general practice. This may be of relevance to other medical schools but also in the postgraduate fields such as on the UK foundation programmes. Further research exploring the impact of a comprehensive range of careers resources may address issues with the limited careers selection in this study. Shaping medical careers is of fundamental importance to all healthcare systems; a trainee changing programmes (or leaving) has significant financial and personal implications. A comprehensive overview of careers interventions — perhaps as a realist or narrative review in the absence of any trials — would seem to be a priority. Routine data such as UKMED could be used to explore the impact of careers interventions, and generate meaningful Level 4 data.^32^ The study has limited implications for an individual clinician with a student, beyond what is already known about the value of enthusiastic role models. However, accessible and relevant resources for students, in addition to careers lectures, do have implications for institutional delivery of careers resources.

Further work within SGUL is underway to expand the specialties included, improve students’ access to the resources across the graduate and undergraduate programmes, and ensure resources are accurate and updated.
